# Use of dietary supplements by individuals with dementia - the caregivers’ perspective

**DOI:** 10.1080/02813432.2025.2496829

**Published:** 2025-04-25

**Authors:** Hilde Risvoll, Marit Waaseth, Kjell H. Halvorsen, Trude Giverhaug, Frauke Musial

**Affiliations:** a NAFKAM, Department of Community Medicine, UiT, The Arctic University of Norway, Tromsø, Norway; b Department of Neurology, NLSH Bodø, Bodø, Norway; c Department of Pharmacy, UiT, The Arctic University of Norway, Tromsø, Norway; d Centre for Elderly and Nursing Home Medicine, Department of Global Public Health and Primary Care, University of Bergen, Bergen, Norway; e Centre for Profession and Quality, University Hospital of North Norway, Tromsø, Norway

**Keywords:** Caregivers, dietary supplements, dementia, patient safety, cross-sectional survey

## Abstract

**Introduction:**

Due to their cognitive challenges, individuals with dementia, often rely on their caregivers for support with activities of daily living. Many of them use dietary supplements (DS) but are at risk of overdosing on DS due to forgetfulness. Moreover, DS may cause adverse events or interactions with prescription drugs (PD). This study aimed to explore the role of caregivers in managing DS use and to identify potential risks associated with its administration.

**Methods:**

A cross-sectional survey among caregivers of individuals with dementia recruited from dementia organisations in North-Norway.

**Results:**

Out of 163 caregivers invited to participate, 104 (64%) participated. Of those, 59 reported that the individual under their care used DS. Twenty-seven caregivers had assisted in DS administration. Eleven expressed concerns around the DS use, and 17 reported that the individual with dementia had previously made mistakes in its administration. Several caregivers did not know whether the individual with dementia used DS nor had difficulties taking DS correctly. Additionally, only 45 were aware that DS use could potentially be harmful. Regarding securing safe use of DS in this patient group, the respondents ranked general practitioners to be most responsible. Nearly 50% of those who had sought advice from health care professionals regarding DS use, were not satisfied with the response. A small sample size and heterogeneous study group must be taken into account when interpreting the results.

**Conclusion:**

Caregivers were often involved in individuals with dementia’s DS use and expressed a willingness to assume responsibility for ensuring safer use. However, several respondents were unaware of the general safety concerns related to DS use. Furthermore, communication with health care professionals regarding DS use, often failed to met the needs of these patients and their caregiver. Collectively, these conditions may impose a risk to individuals with dementia.

## Introduction

Dementia is a medical condition characterised by progressive loss of cognitive function leading to an increasing need for assistance with activities of daily living. Alzheimer’s disease is the most generic form of dementia, with memory problems being the most common symptom [1]. Today, only symptomatic drugs are available for Alzheimer’s disease, although newer antidementia prescribed drugs (PD) not yet licensed in Norway show promising results [2].

Caregivers for individuals with dementia are usually spouses or children. However, other individuals with significant and proximate relationship to the person affected by dementia, may serve as the closest next of kin [3]. In a study from 2015 evaluating the costs from formal and informal care in eight European countries including Norway, the Norwegian caregivers’ annually unpaid care was estimated to €21924 [4]. The World Health Organisation estimate that in high-income countries, dementia-related costs are shared between informal care (45%) and social care (40%) in high-income countries. Whereas, in low- and middle-income countries social care costs accounts for 15% of these costs, while informal care costs accounts for 85% [5]. The need for the caregivers’ assistance will increase with time as the number of individuals with dementia is stipulated to increase as the population ages [6]. The governmental resources cannot keep up with the increasing demand for social care [5]. The Norwegian Dementia Plan 2025 is the Government’s five-year plan (2021–2025) aiming to improve services for individuals with dementia and their families. The Dementia Plan 2025 highlights the caregivers/family members’ important role in the caretaking of individuals with dementia and emphasise the strain this caretaking put on these family members [3].

A challenge that arises early in the progression of dementia, is ensuring the correct administration of PD. If an individual with dementia has problem keeping track of their PD, the same could be the case for their dietary supplements (DS).

DS is defined by The United States (US) Dietary Supplements Health and Education Act (DSHEA) of 1994 as a product meant as a supplement to the diet [7]. DS includes vitamins, minerals, herbs, botanical products, amino acids, or other dietary substances. DS are meant as a supplement to the diet but are often used with the intention to improve specific health conditions such as dementia [8]. Nonetheless, proof of antidementia effect from DS is limited or lacking [9,10]. Due to the risk of interactions with PD, risk of adverse events and lack of proven effect, the Norwegian health authorities have advised against prescribing DS for dementia symptoms since 2019 and encourage health care professionals to uncover such use [11]. Although DS is believed to be ‘natural and safe’ by many, it can cause adverse events and interactions with PD [12,13] such as liver and kidney dysfunctions [14,15], and risk of bleeding [16]. Even lethal cases have been reported [17]. Moreover, cases of illegally added PD to DS have been disclosed, also for DS marketed as cognitive enhancement supplements [18].

Studies have found that up to 50% of individuals with dementia use DS [13,19,20]. Individuals with dementia are often elderly, have other health issues, and are prone to polypharmacy [21]. Due to their memory problems, individuals with dementia can make mistakes with the administration of DS. If they take more than the recommended amount of DS, they are especially vulnerable to adverse events from DS [20]. Interactions between DS and PD have been identified in 11-56% of DS users with dementia [13,20,22]. Individuals with dementia may also forget to take prescribed DS such as e.g. vitamins necessary to supply a documented vitamin insufficiency [23]. Caregivers can be a great support in such situations. A qualitative study among 14 Norwegian general practitioners (GPs) found that several of the GPs had patients with dementia who mixed up DS and PD or stopped taking their PD in favour of a DS without conculting health care personnel [24].

We have studied professional conduct and the attribution of responsibility for individuals with dementia who use DS among health care professionals in home care service and pharmacies, and among GPs [24–26]. Every second home care service employee had been concerned about clients with dementia because of their perceived unsafe use of DS, with one out of four intervening to improve safety [25]. On the other hand, only eight per cent of pharmacy employees had noted potentially unsafe DS use by customers with dementia [26]. Neither employees in home care service, pharmacy employees nor GPs considered themselves as primarily responsible for ensuring the safe DS use among these patients [24–26].

Even though a thorough litterature search on the topic of caregivers’ role as unpaid helpers in securing correct use by individuals with dementia was conducted, we were not able to identify further publications beyond our own study group. We hypothesise that caregivers contribute substantially to safe use of DS by individuals with dementia as demonstrated for PD use [27]. We previously interviewed 151 caregivers and patients about DS use in individuals with dementia [20]. The individuals with dementia often did not initiate the DS use themselves; the initiative was rather taken by their relatives [20]. The same study showed that only 36% of the individuals with dementia received help with the administration of their DS, although 73% received help with the administration of their PD [20].

The data presented here are part of a larger research plan encompassing a series of studies investigating safety aspects related to DS use among individuals with dementia. The overall aim was to investigate the most important target groups involved in the care of home-dwelling individuals with dementia within primary health care. These groups include health care professionals as well as caregivers.

The aim of this study was to explore the caregivers’ involvement in the use of DS among individuals with dementia, including their collaboration with health care professionals and their opinion on attributed responsibility in safeguarding this DS use. Moreover, we wanted to explore any safety issues related to the use of DS by individuals with dementia and the caregivers’ role in ensuring safer use.

## Methods

### Study population and setting

We conducted a cross-sectional survey between September 21^st^ 2017 and July 6^th^ 2018. Respondents were caregivers who were actively involved in their local dementia organisation in North Norway. The Norwegian Health Association’s branch the Dementia Organisation (hereafter named The Norwegian Dementia Organisation) is a national patient interest organisation that aims to improve the conditions for individuals with dementia and their caregivers [28]. The association has local branches in several Norwegian municipalities. Most members are caregivers in addition to patients, health care professionals or others with an interest in the field. The relation between the individual with dementia and their caregiver could vary, nonetheless, the respondents had to define themselves as caregivers. The individual with dementia could be alive or deceased, as we were interested in the caregivers’ experience irrespectively of time. Moreover, many caregivers remained active in the dementia organisation after the individual with dementia had passed away.

Recruitment was facilitated through collaboration with the local branches of the Norwegian Dementia Organisation in 14 municipalities in North Norway. North Norway is an arctic/subarctic geographical area sparsely populated with a few larger towns. The area is 112.986 km^2^ and in 2023 there were 0.49 million inhabitants in this region, or 4.3 inhabitants per km^2^ [29]. Local contacts within the organisation shared information about the study and distributed paper versions of the questionnaire. In order to boost our response rate, we reached out to our contacts up to three times. Local representatives informed us on the number of members attending their meetings. We used this information to estimate the response-rate.

### Questionnaire

No validated questionnaire was available that covered all aspects of interest. We therefore developed a questionnaire specifically for this study based on previous studies from home care services [25] and pharmacies [26]. We conducted a feasibility study on fifteen caregivers to investigate the relevance and readability of the questions and to evaluate the length of the questionnaire. Changes made after this feasibility study included rephrasing of some questions and adding a few response options. The questionnaire covered 29 questions and took 5-20 min to complete depending on whether the individual with dementia had used DS or not, see Supplementary Appendix A.

For the present study, we included a subset of questions covering the following domains:

1. Demographics (gender, type of caregiver, living together with the individual with dementia).
2. Characteristics regarding the DS use by individuals with dementia (use, help with administration, mistakes with the administrations).
3. Caregivers’ attitudes toward DS (personal DS use, beliefs about DS).
4. Attributed responsibility for the safety of individuals with dementia using DS. The respondents were asked to rank who should be responsible for the correct and safe use of DS in individuals with dementia: individuals with dementia themselves, caregivers, GPs, home care services, pharmacies, or DS-retailers. We defined DS-retailers as employees in health food shops, online DS-merchandisers, or complementary and alternative medicine therapists selling DS.
5. Experience with seeking help or advice from health care professionals regarding the individual with dementia’s DS use.

The question about attributed responsibility was ordinal. Respondents were asked to rank the six categories on a scale from 1-6. We merged scores of 2-4 into a medium-level responsibility and scores of 5-6 into a least-responsibility category.

We combined caregivers who did not live together with the individuals with dementia and the few respondents who lived with them periodically, as both these categories were assumed to have less day-to-day knowledge about the individuals with dementia’s situation.

### Ethics

The Regional Committee for Medical and Health Research Ethics presented no objections to the study design (2014/1385). As no patients were included, the project was defined as quality assurance. The survey did not collect personally identifiable information and therefore was not subject to being judged by the Norwegian Data Protection Agency (2017/55343). All respondents were given written information about the study. Submitting the questionnaire was considered as study consent.

### Statistics

We used IBM SPSS (Statistical Package for the Social Sciences) version 29.0 (IBM Corp., Armonk, NY, US) for the statistical analyses.

In this study we used mostly descriptive statistics, as the main aim was to explore any type of safety risk associated with the use of DS. Data are presented as absolute and relative frequencies. Secondarily, we tested for potential differences in characteristics and attitudes between different types of caregivers. Pearson’s chi-square test, Fisher’s exact test and binary logistic regression were used for data analysis. Significance level was set at 5%.

## Results

### Demographics

Out of 163 invited respondents, 104 agreed to participate (response rate 64%). Forty-eight (46%) of the respondents were spouses or other partners (hereafter called partners), 43 (41%) were children and 11 (11%) were relatives. Two persons (2%) did not answer this question. Seventy-nine respondents (76%) were women, 24 (23%) were men, and one (1%) did not answer this question. Fifty respondents (48%) lived or had lived together with the individual with dementia, 47 (45%) had not, and seven (7%) lived together with the individual with dementia in periods (not specified further).

### Characteristics related to DS use

The individuals with dementia had used fatty acids (*n* = 42, 40%), vitamins (*n* = 31, 30%), minerals (*n* = 14, 14%), composite products defined as products containing various products such as herbs, herbal extracts, vitamin, fatty acids, and so forth in combination (*n* = 7, 7%), herbs (*n* = 3, 3%), and six caregivers (6%) did not know which products were used.

Caregivers’ DS use was associated with use by the individuals with dementia. Being worried about the DS use by the individual with dementia was not associated with caregiver’s gender, caregiver’s use of DS, caregiver living together with the person or being aware that DS can cause harm. Being involved in the individual with dementia’s DS use was associated with living together with the individual with dementia. See Table 2 for an overview of statistics.

**Table 1.** Respondents’ answers to questions about DS.

Answers to questions whether the individual with dementia under their care:		Yes	No	Do not know	Item non-responders
	had used DS	59 (57%)	35 (33%)	8 (8%)	2 (2%)
	had trouble taking the DS correctly	17 (28%)	27 (45%)	16 (27%)	1 (1%)
	had taken more DS-tablets than supposed to	1 (6%)	16 (94%)		0
	had taken less DS-tablets than supposed to	5 (29%)	12 (71%)		0
	sometimes took more and sometimes less DS-tablets than supposed to	4 (24%)	13(76%)		0
	had difficulties in differentiating between the different DS	6 (35%)	11 (65%)		0
	had trouble differentiating between DS and PD	5 (29%)	12 (71%)		0
	had received help administering the DS	37 (37%)	18 (17%)	56 (5%)	3 (5%)
Answers to questions whether the caregivers themselves:					
	had used DS	59 (57%)	43 (41%)		2 (2%)
	agreed to the statement that DS may cause harm to users’ health	45 (43%)	17 (16%)	33 (32%)	9 (9%)
	had been worried about the individual with dementia due to safety issues related to that persons’ DS use	11 (18%)	53 (90%)		1 (2%)
	had discussed the individual with dementia’s DS use with health care professionals	12 (20%)	36 (61%)	14 (24%)	3 (5%)
	had been unsatisfied with their communication with health care professionals regarding the individual with dementia’s DS use	5 (42%)	11 (92%)		0
	had been involved in the individual with dementia’s DS use	27 (46%)	25 (42%)		12 (20%)

DS: dietary supplements, PD; prescription drugs.

The percentage of item non-responders is calculated from the total numbers of respondents who were asked to answer this specific question. Some questions were for instance only relevant in cases where the individual with dementia had used DS. In some cases, the total sum exceeds 100% because one or several of the respondents answered no, even if the question was not relevant for them.

**Table 2.** Associations between different characteristics and attitudes in the caregivers, individuals with dementia’s DS use, and the caregivers’ involvement in this use.

			The individual with dementia uses DS		χ2 or Fisher exact test		Binary logistic regression	
Caregivers’ characteristics				(*N* = 59 of 104)					
		N*	Yes	(%)	χ2 or F	p-value	OR	95% CI	
Gender	Female	77	45	(58)	0.01	0.905	0.539	0.158-1.836
	Male	24	14	(58)				
DS use by caregivers	Yes	59	44	(75)	16.07	**<0.001**	**5.013**	**1.948-12.897 **
	No	42	15	(36)				
Lives with the individual with dementia	Yes	50	33	(66)	3.37	0.077	1.835	0.708-4.753
	No	52	26	(50)				
Aware of DS’ potential harm	Yes	44	26	(59)	3.47	0.186	2.380	0.626-8.795
	No	50	26	(52)				
			Caregiver worried about DS use by the individual with dementia		χ2 or Fisher exact test^∞^		Binary logistic regression	
Caregivers’ characteristics				(*N* = 10 of 104)					
		N*	Yes	(%)	χ2 or F	p-value	OR	95% CI	
Gender	Female	47	6	(13)	NA	0.166	0.322	0.063-1.653	
	Male	17	4	(24)					
DS use by caregivers	Yes	46	7	(15)	NA	0.579	1.091	0.207-5.743	
	No	18	3	(17)					
Lives with the individual with dementia	Yes	34	4	(12)	NA	0.495	0.721	0.142-3.602	
	No	30	6	(20)					
Aware of DS’ potential harm	Yes	31	8	(26)	4.04	0.117	4.740	0.700-32.12	
	No	30	2	(7)					
			Caregiver involved in the individual with dementia’s use of DS		χ2 or Fisher exact test		Binary logistic regression		
Caregivers’ characteristics				(*N* = 27 of 104)					
		N*	Yes	(%)	χ2 or F	p-value	OR	95% CI	
Gender	Female	39	20	(51)	0.03	0.873	0.562	0.140-3.126	
	Male	13	7	(54)					
DS use by caregivers	Yes	38	22	(58)	1.47	0.336	1.916	0.361-10.186	
	No	13	5	(38)					
Lives with the individual with dementia	Yes	27	20	(74)	11.04	**0.002**	**9.012**	**2.099-38.704 **	
	No	25	7	(28)					
Aware of DS’ potential harm	Yes	24	11	(46)	0.66	0.578	0.562	0.087-3.628	
	No	25	14	(56)					

Statistically significant results are printed in bold.

*The sum of category N’s per characteristic does not always add up to the total (104) in each of the three comparisons due to missing data.

^∞^F value may not be applicable with the non-parametric Fisher exact test, especially when the p-value is not significant.

### Help with the DS use

Twenty-seven of the respondents reported that they had been involved in the patients DS use in several ways. Three individuals (one partner and two children) had given advice. Twenty-one individuals (eighteen partners and three children) had given practical assistance (not further specified), and eleven (five partners, four children and two other relatives) had supplied the person with dementia with information about DS. According to the respondents, thirty-eight of the fifty-nine individuals with dementia who used DS (64% of the DS users) had received assistance with administering the DS. Thirty of the respondents said that they had helped with the administration of DS (twenty-four partners and six children).

### Attributed responsibility

**Figure 1.**
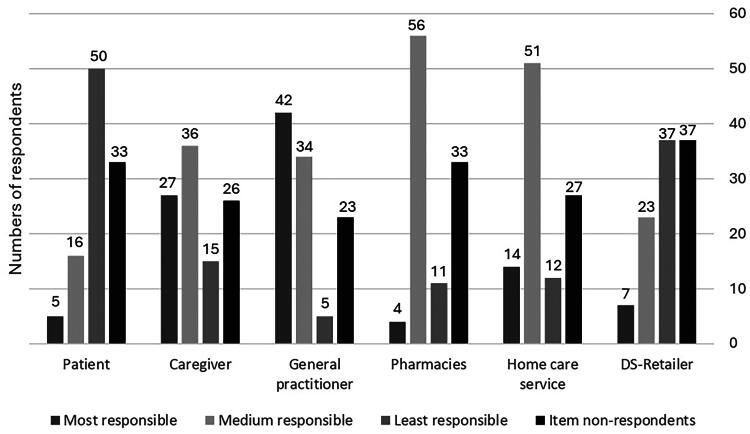
The respondents’ ranking of responsibility for the safety of individuals with dementia who use DS. DS, dietary supplements. For the question ‘Where should the responsibility for the safe use of DS in individuals with dementia be placed?’ respondents were asked to rank the six categories from 1 (most responsible) to 6 (least responsible). We merged ranks 2–4 into medium-level responsible and ranks 5–6 into least responsible. DS-retailers could be health food store staff, internet retailers, complementary and alternative medicine therapists selling DS.

The caregivers ranked GPs as the most responsible and themselves as the second most responsible for ensuring safe use of DS for the individuals with dementia. The individuals with dementia themselves were ranked as least responsible, see Figure 1.

### Experience with health care professionals

Twelve caregivers had consulted health care professionals about the use of DS by the individual with dementia (20% of the cases where the individual with dementia used DS). The use was discussed with a GP (*n* = 7), a pharmacist (*n* = 2), or an employee in home care service (*n* = 3), and two discussed the issue with an unspecified health care professional. In seven cases more than one type of health care professional was consulted. The conclusions from these consultations were that the use was safe and could continue (*n* = 5), it was unsafe and had to be stopped (*n* = 1), or it was considered safe if the individual with dementia received help with the administration (*n* = 6). Two received other types of advice, not further specified, and four respondents did not know the conclusion of the consultation.

Forty-two per cent of those who consulted health care professionals (*n* = 5) described their communication with health care professionals about this topic as unsatisfactory. Two reported that their questions were not answered, or their concern was not taken seriously. In three cases the health care professionals did not check for interactions as requested, and in two cases the health care professionals showed a condemning attitude towards the DS use. It was possible to choose more than one answer to this question. The total numbers are small, and there was no statistically significant difference between the type of advice and experience of unsatisfying communication (data not shown).

### Other comments

The questionnaire included a box for other comments. Eight out of nineteen comments expressed that there was too much dubious marketing for DS products. The GPs role as a consultant regarding DS use was emphasised by two respondents. Another two respondents emphasised the importance of measuring blood levels of vitamins. The remaining comments were details about their own experience.

## Discussion

### Main findings

More than every second respondent reported that the individual with dementia under their care had used DS. The caregivers often assisted the individual with dementia with the administration of DS and finding information about DS, yet one-third of the individuals with dementia did not receive any help. Moreover, every fourth caregiver reported that the individuals with dementia made errors administering their DS, and one-fifth of caregivers expressed worries about their safety related to this DS use. Several caregivers were unaware of whether the individual with dementia under their care used DS, nor if there were any associated challeges with such use. More than one-third of caregivers who consulted health care professionals for advice about this use were not satisfied by the response.

### Findings related to other studies

DS have an ambiguous role in medical practice due to a dual role, functioning as a diet [7] and as a supplement the patients take at their own discretion to promote health [8]. DS are used by people to improve dementia symptoms despite limited evidence of their effectiveness, and despite concerns about safety aspects regarding this usage [20]. Fifty-seven per cent of the caregivers in this study reported that the individual with dementia under their care had used DS. DS include a wide variety of products, from vitamins used for a proven deficiency, to herbs or composite products with higher risk of interactions with PD or adverse events [7,13,19,30]. This study does not separate between different forms of DS. In general, individuals with dementia are, due to their cognitive challenges, at risk of making mistakes with the administration of DS, such as either taking too few vitamins for a proven deficiency or taking too much of a DS-product that may harm them. Twenty-eight per cent of the respondents in this study reported that the individual with dementia under their care had made mistakes with the administrations of their DS, and 18% had been worried that the DS use could harm the individual with dementia. Even though the design of this study did not allow us to detect interactions and adverse effect from DS, these were found in 11% of DS users with dementia in a former study by our group [20].

This study investigateed caregivers role in assisting individuals with dementia with their use of DS, with the aim to avoid harm from this use. So far, we were not able to identify other studies exploring caregivers’ role in the correct administration ofDS, regardless of supplement type. Of some relevance, were two studies related to nutritional deficits. One Finnish study demonstrated that male gender in caregivers corresponds to lower nutritional status of patients with dementia [31]. A Swedish qualitative study among 17 caregivers revealed that unexperienced food providers (all men) lacked knowledge on how to shop for and prepare nutritious food, and therefore needed support to perform this task [32]. In this Swedish study all participants were recommended vitamin DS by their GPs. No differences regarding caregivers gender were found in our study, although this may be due to low statistical power.

A high proportion of the caregivers reported that they would like to take responsibility for the safety concerning the use of DS by the individuals with dementia under their care. They ranked themselves second to GPs in terms of responsibility, and 25% of the respondents (*n* = 27) believed that caregivers should take full responsibility, Figure 1. On the other hand, only four per cent of home care service employees (*n* = 231) and two per cent of pharmacy employees (*n* = 105), who were asked the same question in former studies, were willing to take full responsibility for these individuals’ safety [25,26]. Even though many of the caregivers were willing, there are several reasons why the caregivers may face challenges taking this responsibility. Only 43% of the caregivers knew that some DS might cause harm to users’ health, which corresponds to 49% of 151 caregivers in a former study [20]. Caregivers are not obliged to have knowledge about interactions and adverse events from DS or PD, or to know whether the patient’s health condition, such as a possible liver- or kidney failure, makes them more vulnerable to certain types of DS. In addition, caregivers themselves may have cognitive impairments as this is a common problem, especially among older adults [6]. As less than half of the caregivers knew that DS may cause harm to users’ health, they may not see a need to assist the individual with dementia. Some caregivers were not aware whether the individual with dementia under their care used DS, and they did not know about safety issues related to the use (such as issues with correct administration). For these reasons, this study may in fact underestimate the problematic use of DS by individuals with dementia in the study population.

About half of the caregivers in this study assisted the individual with dementia’s DS use. This assistance was done in several ways, such as giving advice, finding information, communicating with health care professionals, and helping with the administration. We have in a former study demonstrated that individuals with dementia who live alone, are less likely to receive help administering DS (*n* = 151) [20]. Similarly, in this study, being involved in assisting the DS use, was associated with living together with the individual with dementia. This is in line with a study among 100 Spanish caregivers of individuals with dementia, demonstrating that 29% of these patients did not adhere to their chronic PD treatment. Adherence was increased when the caregivers were first degree relatives (spouse or child) and female living together with the person with dementia. Caregivers considered the use (90%) and administration (91%) of the PD treatment easy or very easy to handle in their daily life [27].

The Norwegian Dementia Plan 2025 does not mention DS or assistance with DS administration but emphasises the importance of the caregivers’ contribution in the care of individuals with dementia [3]. Many caregivers feel overlooked and underappreciated, despite holding vital knowledge about the individual with dementia that is crucial for healthcare services. Furthermore, the caregivers are often left to coordinate professional services themselves. One-half to two-thirds of the caregivers are experiencing stress and poorer health due to the total burden of care [3]. Thus, taking responsibility for the individual with dementia’s DS is time consuming and likely a burden. Therefore, it is important to be aware of the total burden for the caregivers, as the Norwegian Dementia Plan emphasises [3]. Contrary to PD administration, where one would expect assistance and clear guidelines from health care professionals, little or no assistance might be expected regarding DS administration. This study and a former qualitatively study of 14 Norwegian GPs [24], show that answers and support from GPs about DS use, might not be so readily available as one might expect.

The caregivers assigned the GPs the greatest responsibility for safe use of DS by these patients. In earlier studies, employees in home care service (*n* = 231) [25] and pharmacies (*n* = 105) [26] expressed the same opinion. Interestingly, none of the fourteen GPs interviewed in a previous qualitative study wanted to take on this responsibility because they did not have the necessary prerequisites or tools to consider if the DS was safe or not [24]. Instead, they referred to the caregivers and health care professionals in home care service as responsible. In conclusion, in a situation where nobody accepts responsibility for the safe use of DS in individuals with dementia, it is left to the individuals with dementia themselves or their caregivers.

In 2019, shortly after this study was conducted, the Norwegian health authorities, in their guidelines for dementia treatment, advised against prescribing DS for dementia symptoms, and encouraged health care professionals to uncover such use to avoid interactions with PD [11]. However, to our knowledge, there has not been a public campaign, and it remains unclear how well known this recommendation is. In the field of securing safe use of DS in patients with dementia, we believe it is particularly important to include the caregivers. Moreover, we would support strategies to implement this systematically. In our view, it is important to include caregivers in medication reconciliation, and that this reconciliation should also include DS. Moreover, we emphasize the importance of guiding the caregivers in evaluating the use of DS with the aim to avoid risk. DS can be of importance when it comes to nutrition, but ideally vitamins and other nutrients should be provided by nutritious and sufficient food, as the Norwegian Dementia Plan 2025 suggests.

For health care professionals, such as GPs, to take responsibility for safe use of DS by individuals with dementia, three prerequisites are needed; knowledge about safety challenges from DS in general, knowledge about cognitive problems and dementia in their patients and knowledge about use of spesific DS by their patients with dementia [24]. When it comes to the second and third prerequisites, caregivers play a central role. The Dementia Plan highlights the challenges of underdiagnosing of dementia in Norway [3]. To date, only 45,000 individuals are registered with dementia in Norway while it is stipulated that the correct number is around 100,000 [6]. Some of these under-diagnosed dementia cases might not receive the help needed with activities of daily living such as administrating PD or DS. This underdiagnosing can be improved by more focus on openness and by increasing the knowledge about dementia symptoms in the general population, and the caregivers may be crucial for communicating this symptoms to health care professionals [3].

When it comes to knowledge about patients with dementia’s DS use, previous studies have shown that health care professionals are often unfamiliar with this use [19,30]. We believe caregivers can assist individuals with dementia by helping them communicate with health care professionals about this topic, but caregivers must be aware of the need for this communication. Some of our respondents had communicated with health care professionals, but almost half of those who had, found this communication problematic because the health care professionals condemned the use, did not check for interactions, or did not respond to their questions. None of the 14 Norwegian GPs in the above-mentioned qualitative study, mentioned conflicts with caregivers about DS use [24]. However, several of the GP’s said it was difficult to answer questions about DS because of the lack of evidence on effect and safety. One GP refused to answer questions or discuss DS with patients because of this. The practice differed among the GPs [24]. The results align with the caregivers’ experience about communication but also explain how the lack of studies and the regulatory condition on DS hinders the GPs from meeting the patients’ or/caregivers’ needs.

Harris et al. found that doctors’ bias can hinder communication about DS in many ways and make their patients reluctant to disclose their DS use [33]. Ascertainment bias occurs when a clinician’s thinking is shaped by prior expectations. When collecting patients’ medication histories, allopathic clinicians rarely ask patients which DS they use, and patients often do not report using them [33]. Not asking or showing interest in DS is the most common reason for non-disclosure. Likewise, health care professionals’ negative feelings may influence their decision making. Some health care professionals have negative attitude towards DS, and this may be sensed by the patients, making them reluctant to disclose such use or ask for advice. In a former qualitative study, 14 Norwegian GPs believed their patients could be reluctant to discuss DS use with them because they were afraid of ‘being blamed, ridiculed, not being heard, or believing that the topic was not relevant’ [24]. These GPs expressed that the topic DS seldom came up in consultations, however their approach varied. The one GP who asked systematic about DS believed that 50% of her older patients used DS, contrasting the one GP who never asked about DS and believed that only 5-10% used DS [24]. In an observation study in California in 2013 Tarn found that DS was discussed in one out of four consultations in primary health care [34] indicating that this is a topic patients wants to discuss with their GPs.

A few of our respondents provided open comments in the questionnaire, and more than half of these highlighted concerns about the misleading marketing of DS. In a previous qualitative study, several of the included Norwegian GPs raised similar concerns [24]. Economic exploitation of the individuals with dementia was also a concern mentioned by the GPs in that study [24]. While the present survey did not specifically delve into commercial interests or the economic impact on patients, we acknowledge the ethical considerations related to DS marketing directed toward individuals with dementia or their caregivers. Health claims about dementia have for instance been found in web advertising where DS were described as ‘effective, natural, powerful, strong, dependable, pure or of high quality’ [35]. The Norwegian Food Safety Authority found illegal health claims in nine out of ten commercials for DS in a report from 2022 [36]. Health claims on DS is illegal in Norway, but the enforcement of this regulation has been weak. Nevertheless, The Norwegian Food Safety Authority focus on increasing the control in a report from 2024 [37]. A Polish study from 2022 similarly found extensive use of health claims and persuasion methods in commercials about DS products [38]. A significant negative relationship has been found between knowledge about DS and trust in DS marketing [39]. The same study found limited knowledge about DS in the 220 non-medically educated respondents.

### Strengths and limitations

The response rate of 64% was acceptable. The data represents new knowledge in a field where little is known before and initial mapping of the problem is necessary. However, the design allows some comparison with former studies from our group, for instance the comparison between this study population recruited from the community and a study population recruited from specialised health care service [20].

Major limitations are small number of respondents, recruitment from a limited geographical area and a heterogen study group.The sample size comprises a limited number of caregivers of patients with dementia who used DS. Among them are partners, adult children, and other relatives, of whom some did not cohabite with the patient. We did not collect data about the individual with dementia and their dementia diagnosis. We only know that their dementia symptoms were severe enough for their caregivers to be motivated to join The Norwegian Dementia Organisation. The material does not specify whether the patients are deceased or alive at the time of inclusion, nor does it differentiate between institutionalised and non-institutionalised patients. However, we asume all of these patients had been home dwelling initially. The small number of respondents in this study limites the investigation of differences between subgroups.

The geographical area of data collection is restricted to 14 municipalities in North Norway. One should bear in mind that attitudes towards DS and towards caretaking of family members with dementia varies between cultures. Also, caregivers recruited from The Norwegian Dementia Organisation may not be representative, as not all Norwegian caregivers of individuals with dementia are active members of this organisation. Members may be particularly interested in defeating dementia. However, the organisation states no official policy regarding DS, and we have therefore no reason to attribute certain attitudes towards DS to the members. We believe this topic about patient safety is generic interest. Even so, we cannot exclude that the members who answered the questionnaire might be particularly interested in DS.

The questionnaire was not validated. All data were based on self-reports from our respondents regarding their own actions or on the actions of the individual with dementia, (third party). Therefore, the data may be influenced by subjectivity, recall bias and lack of avareness of DS use. The response option ‘Do not know’ was given when the respondents were asked about the actions of the individual with dementia. The questionnaire had up to 29 questions, this could be difficult for an elderly caregiver to complete, especially if it required recalling events from the past. Some of the caregivers did not live together with the individual with dementia and might therefore not know so much about their DS use. We therefore assume underestimation of problems related to these patients’ DS use is more likely than overestimation.

Some of the questions appearing late in the questionnaire, such as the question whether the respondents ‘had been involved in the individual with dementia’s DS use’, had a higher number of item non-responders than questions appearing early in the questionnaire. There were however no objections to the length of the questionnaire in the feasibility study or from our patients’ research partners, see Acknowledgement, and any uncertainty in the wording that was discovered in the feasibility study were corrected.

The proportion of item non-respondents to the question of attributed responsibility is high, see Figure 1. This is probably a question the respondents have never thought about before, and they might not have an established answer. The definition of DS in this study is broad, and this could have made it more difficult for the respondents to answer this question, as one could perceive the responsibility different if the DS used was a prescribed vitamin D for a confirmed deficiency, than if it was an herbal mixture bought online.

As there are very few studies on this topic, it is not possible to compare this study to similar studies from other study-group, which limits the discussion. We have however tried to minimise confirmation bias by listening to different experts, including our patient research partners (see acknowledgement), and the respondents in our feasibility study when designing the study and the questionnaire. Because of the limited number of available studies, we have, in the overall project, investigated this problem from several different perspectives [20,24–26]. Larger and more generalisable studies would be highly appreciated.

## Conclusion

This survey was conducted to detect safety issues related to the use of DS by persons with dementia exploring the caregivers perspective. The safety issues identified have not been described before and add to our knowledge about risk associated with DS use in individuals with dementia. This study was not design for gaining sufficient data to generalize how often there are reasons for concern in different subgroups of persons with dementia according to what type of DS they were using. Larger studies are needed to investigate this. Nonetheless, we find it of importance that caregivers experienced concern about this DS use and furthermore that they were not always able to get the assistance or support that they needed from health care personnel.

A high proportion of the caregivers assisted the individuals with dementia in their DS use to make the use safer. Although most caregivers assigned the GPs the highest responsibility for these patients’ safety, one of four were willing to take full responsibility themselves.

Caregivers not living together with the individual with dementia were less involved and may not be aware of these person’s DS use. Health care professionals should thus be extra concerned about the status of DS use among individuals with dementia living alone, as potentially unsafe DS use may remain concealed. There is a need for more quality ensured information about the risks from improper DS use, both for individuals with dementia and their caregivers. We suggest that Norwegian GPs follow Norwegian health authorities’ recommendation and start systematically discovering use of DS by individuals with dementia, involving the caregivers to secure information about which DS is used and whether the administration of DS is safe for these persons. Finding reliable information on every DS in use can be difficult, but without an interest in finding out more, this safety problem will not be properly addressed.

## Supplementary Material

Appendix A Questionare.pdf

## Data Availability

The datasets used and analysed during the current study are available from the corresponding author upon reasonable request.

## Abbreviations

DS: dietary supplements
PD: prescription drugs
GP: general practitioner.
