# Supplementary material for: Use of dietary supplements by individuals with dementia - the caregivers’ perspective
Source: Scand J Prim Health Care. 2025 Apr 25;43(3):672–83. doi: 10.1080/02813432.2025.2496829 (PMC12377123; doi:10.1080/02813432.2025.2496829)
Supplement: Appendix A Questionare.pdf [file IPRI_A_2496829_SM8495.pdf]

Use of dietary supplements by persons with dementia - The caregivers perspective

We ask you to participate in this survey because of your role as a caregiver to a person with dementia. You can give answers even though the person with dementia is deceased. The survey aims to explore the safety of persons with dementia who use dietary supplements/natural products. Further on, the survey wants to explore whether the caregivers are involved in this use. Dietary supplements/natural products may contain different vitamins, minerals, fatty-acids, or a combination of several substances. The supplements/products could have been bought online, in health store, in grocery shops, in pharmacies or directly from a therapist.

1. Gender
   a. Female
   b. Male
2. Relation to the person with dementia
   a. Spouse/partner
   b. Child/son-or daughter in law
   c. Other type of relation (grandchild, sibling, niece, nephew)
3. Do or did you live together with the person with dementia
   a. Yes
   b. No
   c. Only in periods
4. Do you use dietary supplements. By dietary supplement one understand all types of vitamins, minerals, fatty-acids, herbs or combinations of these
   a. Yes
   b. No
5. Do or did the person with dementia use dietary supplements/natural products?
   a. Yes
   b. No
   c. Do not know

If you choose “no” as a response to question 5, you may skip questions 6 up to 26, and answer questions 27, 28 and 29. If you respond, «do not know», you may evaluate if any of the questions 6-26 are of relevance for you. Please skip questions without relevance. If you answer “yes,” most of the questions are of relevance for you.

6. If the person with dementia no longer use dietary supplements/natural products, when did the use take place?
   a. The person still use dietary supplements/natural products
   b. Less than a month ago

c. From one to three months ago
d. From three months to one year ago
e. More than one year ago
f. Do not know

7. Which type of dietary supplements/natural products are/were used? You can choose more than one option.
   a. Vitamins
   b. Minerals
   c. Fatty-acids (omega-3 or similar)
   d. Herbs
   e. Composite products (mixture of vitamin, minerals, fatty-acids and/or herbal products)
   f. Do not know
8. Were you ever worried about the use of dietary supplements/natural products by the person with dementia? (unsure whether the products in use were safe, unsure whether the products could be used together with the persons prescribed drugs, afraid the person did not manage to take the products correctly, et cetera).
   a. Yes
   b. No
9. Did the person with dementia have problems taking the dietary supplements/ natural products correctly (correct dose at correct time, not mix up different products)?
   a. Yes
   b. No
   c. Do not know

Answer question 10 only if you answered “yes” to the previous question

10. In which way did the person with dementia have problems taking the dietary supplements/natural products correctly? You can choose more than one alternative.
    a. Took more tablets than supposed to
    b. Took less tablets than supposed to
    c. Sometimes took more, sometimes took less tablets than supposed to
    d. Did not manage to separate the different dietary supplements/natural products from each other
    e. Mixed up dietary supplements/natural products and prescription drugs
11. Have the person with dementia received help taking the dietary supplements/natural products correctly?
    a. Yes
    b. No
    c. Do not know

Answer the next question only if the person with dementia did receive help taking the dietary supplements/natural products correctly

12. Who helped the person with dementia taking the dietary supplements/natural products correctly?
    a. Me
    b. Other relatives
    c. Home care service
13. Did the person with dementia have any positive effects from the dietary supplements/natural products?
    a. Yes
    b. No
    c. Do not know

14. If there were a positive effect, what effect?
15. Did the person with dementia who used dietary supplements/natural products experience any negative effects from these products (adverse events, interactions with prescription drugs)
    a. Yes
    b. No
    c. Do not know
16. If there were negative effects from this use, can you describe these negative effects (dizziness, rash, stomach aches, other symptoms), and name any prescription drugs with which there were a negative reaction.
17. Do you know whether the person with dementia or the caregivers have received information about the dietary supplement/natural products in use
    a. Yes
    b. No
18. Was the use of dietary supplements/natural products discussed with health care personnel?
    a. Yes
    b. No
    c. Do not know

You only need to answer questions 19-24 if you answered “yes” to question number 18

19. Which type of health care personnel were the use of dietary supplements/natural products discussed with. You may choose more than one option.
    a. Employees in pharmacy
    b. Employees in home care service
    c. A general practitioner
    d. A medical doctor at a hospital
    e. A nurse at a hospital
    f. Other types of health care professionals (clinical nutritionists, physiotherapists, psychologists, health secretary, et cetera)
20. Whom gave the best advice? You can only choose one alternative.
    a. Employees in pharmacy
    b. Employees in home care service
    c. A general practitioner
    d. A medical doctor at a hospital
    e. A nurse at a hospital
    f. Other type of health care professionals (clinical nutritionists, physiotherapists, psychologists, health secretary, et cetera)
    g. I do not know
21. Which advice were given? You can only choose one alternative.
    a. The use of dietary supplements/natural products was safe and could be continued
    b. The use of dietary supplements/natural products was unsafe and should be stopped.
    c. The use of dietary supplements/natural products was safe if the person with dementia received help with the administration
    d. Other types of advice
    e. I do not know
22. Was the advice followed?
    a. Yes
    b. No
    c. I do not know

23. Did you experience unsatisfactory communication with health care personnel regarding the use of dietary supplements/natural products by the person with dementia in your care?
   a. Yes
   b. No
24. In what way was the communication unsatisfactionary? You may choose more than one option.
   a. I experienced rejection from health care personnel. The health care personnel did not want to discuss the use of dietary supplements/natural products.
   b. The health care personnel did discuss the use of dietary supplements/natural products but did not check whether the use were safe as requested.
   c. The health care personnel did not help the person with dementia to a safe administration of dietary supplements/natural products.
   d. The health care personnel condemned the use of dietary supplements/natural products.
25. Have you been involved in the use of dietary supplements/natural products by the person with dementia
   a. Yes
   b. No

Answer the next question only if you answered “yes” to the previous question

26. In which way have you been involved in the use of dietary supplements/natural products by the person with dementia? You may give more than one answer.
   a. Given advice (initiated the use of dietary supplements/natural products, encouraged to end the use of dietary supplements/natural products et cetera).
   b. Helped in a practical way (bought dietary supplements/natural products for the person with dementia, helped securing correct administration et cetera).
   c. Gained information about specific dietary supplements/natural products to support the person with dementia (read pamphlets or other lectured, looked up information online, asked health care personnel et cetera).
27. Who do you think should be responsible for the safe use of dietary supplements/natural products by home-dwelling persons with dementia? Prioritize from 1-6, by prioritizing1 for the category/option you believe to be most responsible, 2 for the second most responsible, et cetera, until you prioritize the least responsible category 6. This is a question without a collective understanding, so we want to learn the caregivers’ opinion. The options are presented in a random order, and the order will vary between different participants to avoid influencing the prioritizing.
   a. Caregivers
   b. Retailers of dietary supplements/natural products
   c. Pharmacies
   d. General practitioners
   e. The person with dementia themselves
   f. Home care service
28. Do you agree with the following statement:” Use of dietary supplements/natural products may cause harm to user’s health”?
   a. Yes
   b. No
   c. Do not know
29. Are there any important topics missing in this survey? Do you want to add any comments?
